# Application of silicon nanoparticles in agriculture

**DOI:** 10.1007/s13205-019-1626-7

**Published:** 2019-02-18

**Authors:** Anshu Rastogi, Durgesh Kumar Tripathi, Saurabh Yadav, Devendra Kumar Chauhan, Marek Živčák, Mansour Ghorbanpour, Nabil Ibrahim El-Sheery, Marian Brestic

**Affiliations:** 1 Meteorology Department, Poznan University of Life Sciences, Piątkowska 94, Poznań, 60-649 Poland; 2 Amity Institute of Organic Agriculture (AIOA) Amity University, Noida, 201313 India; 3 Department of Biotechnology, Hemvati Nandan Bahuguna Garhwal (Central) University, Srinagar Garhwal, Uttarakhand 246174 India; 4 D D Pant Interdisciplinary Research Laboratory, Department of Botany, University of Allahabad, Allahabad, India; 5 Department of Plant Physiology, Slovak University of Agriculture, A. Hlinku 2, Nitra, 94976 Slovakia; 6 Department of Medicinal Plants, Faculty of Agriculture and Natural Resources, Arak University, Arak, Iran; 7 Agricultural Botany Department, Faculty of Agriculture, Tanta University, Tanta, Egypt

**Keywords:** Silicon, Mesoporous, Nanocarrier, Fertilizers

## Abstract

The beneficial effects of silicon and its role for plants are well established; however, the advantages of silicon nanoparticles over its bulk material are an area that is less explored. Silicon nanoparticles have distinctive physiological characteristics that allow them to enter plants and influence plant metabolic activities. The mesoporous nature of silicon nanoparticles also makes them good candidates as suitable nanocarriers for different molecules that may help in agriculture. Several studies have shown the importance of silicon nanoparticles in agriculture, but an overview of the related aspects was missing. Therefore, this review brings together the literature on silicon nanoparticles and discusses the impact of silicon nanoparticles on several aspects of agricultural sciences. The review also discusses the future application of silicon nanoparticles in plant growth, plant development, and improvement of plant productivity.

## Introduction

**Fig. 1:**
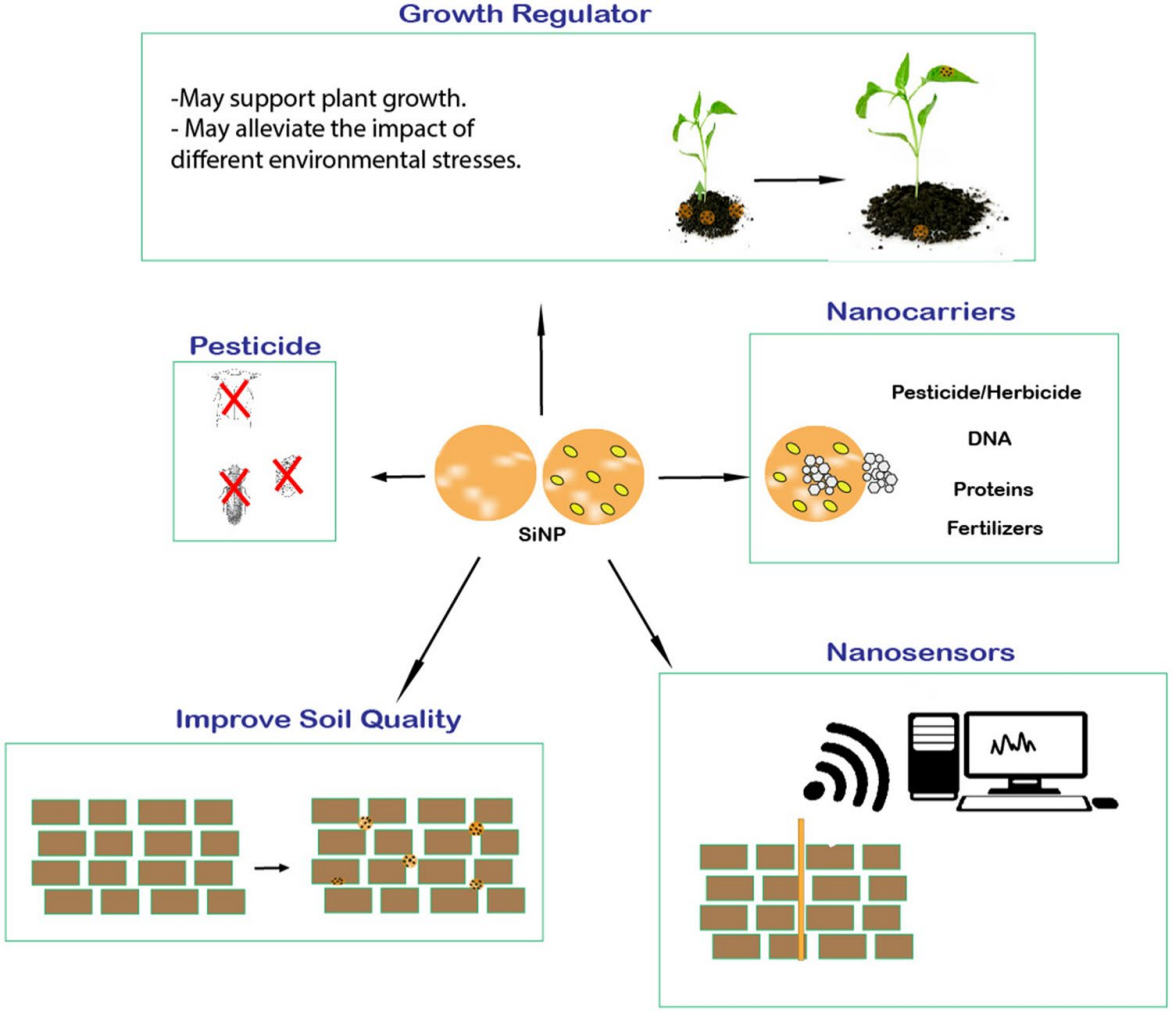
Si-NP in agriculture: The illustration presents the potential use of Si-NP in agriculture

A metalloid is an element with intermediate physical and chemical properties between those of metals and nonmetals. Among the different metalloids, silicon is the most common on earth and is the second most abundant element in the earth’s crust after oxygen (Epstein 1994). Silicon is also considered somewhere between an essential and nonessential element for plants, as it is not required for the survival of most plants, but plants benefit and are better adapted to different environmental stress conditions in the presence of silicon (Epstein 1994; Luyckx et al. 2017). Silicon has also been observed to be used by plants to strengthen their cell walls; the plants of the *Equisetaceae* family cannot survive in nutrient solutions lacking silicon (Epstein 1994). Therefore, silicon is considered an essential element for the *Equisetaceae* family (Epstein 1994). Silicon content in plants was observed to vary from 0.1 to 10%, which was attributed to different mechanisms of silicon uptake (Liang et al. 2007). Dissolved silicon was reported to be absorbed by plants in the form of monosilicic acid, and in some plants with a high accumulation capacity of metalloids, different silicon transporter genes (such as LSi1, LSi2, and LSi6) have been reported to help in its transportation (Rao and Susmitha 2017). Nanoparticles may exhibit different properties than their bulk material due to their small size, greater surface area-to-weight ratio, and different shapes (Roduner 2006). Similarly, silicon nanoparticles (Si-NPs) were observed to exhibit different physical and chemical properties than their bulk material (O’Farrell et al. 2006). Therefore, it is important to know how differently Si-NPs interact within the environment. Due to their unique properties, Si-NPs exhibit great potential in agriculture and may work better in alleviating different abiotic stresses than bulk material (Tripathi et al. 2015, 2017; Cui et al. 2017; Abdel-Haliem et al. 2017). Apart from the direct impact of Si-NPs on plant growth and development, Si-NPs can also be used as nanopesticides, nanoherbicides, and nanofertilizers (Fig. 1). Silicon nanoparticles may also be used as delivery agents for proteins, nucleotides, and other chemicals in plants; nanozeolite and nanosensors incorporate Si-NPs and may be effectively used in agriculture for increasing the water retention of soil and for soil monitoring, respectively (Fig. 1). The focus of this review is to systematically present the potential of Si-NPs in agriculture.

In the following sections, we will discuss the direct and indirect impact of Si-NPs on plants and discuss their importance in agriculture.

## Direct impact of Si-NPs on plants

The response of plants to nanoparticles depends on various factors, including the size, shape, method of application, chemical properties, and physical properties of the nanoparticles (Rastogi et al. 2017). Recent studies have shown that Si-NPs may directly interact with plants and impact their morphology and physiology in various ways, including the addition of structural color to the plants, and help in improving plant growth and yield (Bao-shan et al. 2004; Strout et al. 2013; Suriyaprabha 2014; Siddiqui and Al-Whaibi 2014). Some studies have also indicated a negative impact of Si-NPs on plants (Slomberg and Schoenfisch 2012; Le et al. 2014). To provide a better understanding, important studies involving plants and Si-NPs are summarized in Table 1.

**Table 1:** Impact of Si-NPs on plants, where (–) indicates data are not available

Composition; origin; size (nm)	Concentration, treatment	Composition; origin; size (nm)	Plant	Impact (in comparison to control group)	References
SiO_2;_ commercial; (–)	62, 125, 250, 500, 1000 µL/L, seedling soaked with NP solution	SiO_2_; commercial; (–)	*Larix olgensis*	500 µL/L showed the best results with an increase in mean height, root length, number of lateral roots, and chlorophyll concentration	Bao-shan et al. (2004)
Nano-Si; commercial; (–)	1, 2 mM, seeds germinated in Petri plate	Nano-Si; commercial; (–)	*Lycopersicum esculentum*	1 mM NPs were observed to act better (in comparison to 2 mM) in the adaptation of plants under salinity stress, with improvements in root and shoot growth	Haghighi et al. (2012)
Nano-Si; commercial; 20–35	1, 2 mM, seedlings though nutrient solution	Nano-Si; commercial; 20–35	*Solanum lycopersicum*	Si and Si-NP alleviated the effect of salinity stress on the fresh weight, chlorophyll concentration, photosynthetic rate, and leaf water content of the plant 1 mM Si-NPs were observed to dramatically increase the photosynthesis rate and transpiration under 50 mM salinity stress No significant differences in the other characteristics were observed between Si-NP and Si	Haghighi and Pessarakli (2013)
SiO_2_; biological; 20–40	15 kg/ha, soil in field	SiO_2_; biological; 20–40	*Zea mays*	Si-NP-treated maize was observed to contain higher silica than micro-Si-treated maize or the control Root elongation was observed to be significantly increased	Suriyaprabha et al. (2013)
SiO_2_; commercial; < 50	1 mM, seeds germinated in Petri plate	SiO_2_; commercial; < 50	*Lens culinaris*	NP improves the germination and early growth of plants under salinity stress	Sabaghnia and Janmohammadi (2015)
SiO_2_; commercial; 12	2,4,6,8,10,12,14 g/L, seeds germinated in Petri plate	SiO_2_; commercial; 12	*Lycopersicum esculentum*	8 g/L was observed to significantly enhance seed germination, mean germination time, seed germination index, seed vigor index, seedling fresh weight, and dry weight	Siddiqui and Al-Whaibi (2014)
Si-NP; (–), (–)	10 ml/L, sprayed on plant	Si-NP; (–), (–)	*Ocimum basilicum*	NPs alleviated the impact of salinity stress	Kalteh et al. (2014)
SiO_2_; commercial; 10–30	10, 50, 100 mg/L, seedling through irrigation	SiO_2_; commercial; 10–30	*Crataegus aronia*	A dose-dependent impact on alleviating drought stress was observed by increases in plant growth properties and photosynthetic pigment concentrations and decreases in xylem water potential and MDA content	Ashkavand et al. (2015)
Nano-Si; commercial; (–)	1.5, 3 mM, seeds grown in pot	Nano-Si; commercial; (–)	*Vicia faba*	Si-NPs were observed to slightly improve flowering when compared with Si or the control	Roohizadeh et al. (2015)
Nano-Si; chemical; 75–125	10 µM, seed and seedlings, in petri plate or hydroponic	Nano-Si; chemical; 75–125	*Pisum sativum*	Addition of Si-NPs together with Cr(VI) was observed to protect pea seedlings against Cr(VI) phytotoxicity NPs were observed to upregulate the antioxidant defense system in the presence of Cr(VI)	Tripathi et al. (2015)
Mesoporous nano-Si; chemical; 20	200, 500, 1000, 2000 mg/L, seed and seedlings, in Petri plate or hydroponic	Mesoporous nano-Si; chemical; 20	Wheat Lupin	NPs facilitated photosynthetic activity and plant growth NPs were observed to be accumulated in different parts of treated plants following root uptake	Sun et al. (2016)
Nano-Si; biological; (–)	7.5 g/pot, seeds in pot with soil and NP	Nano-Si; biological; (–)	Rice	The expression rate of the silicon uptake genes Lsi1 and Lsi2 increased in nano-Si in comparison to the control, but was less than that in plants treated with Si ions Overall, no positive/negative impact of nano-Si on plants was observed under salinity stress	Abdel-Haliem et al. (2017)
Nano-Si; chemical; 19, 48, and 202	1 mM, tissues, NP added in culture solution	Nano-Si; chemical; 19, 48, and 202	Rice	Under cadmium toxicity, the survival of rice cells was observed to be dependent on the size of NPs Gene expression for Si uptake (OsLsi1) and Cd transport to vacuoles (OsHMA3) was observed to be upregulated The expression of Cd uptake genes (OsLCT1 and OsNRAMP5) was observed to be downregulated	Cui et al. (2017)
SiO_2_; commercial; 20–30	0-2.5 mM, seedlings, hydroponic	SiO_2_; commercial; 20–30	*Trigonella foenum*	Can modulate the PST transporter and, therefore, can increase the translocation of NPs/Si NPs influenced Si uptake, translocation and accumulation, the lignification of cell walls, and the formation of stress enzymes in comparison to the control or sodium silicate	Nazaralian et al. (2017)
Nano-Si; chemical; 75–125	10 µM, seedlings, hydroponic	Nano-Si; chemical; 75–125	*Triticum aestivum*	Si and Si-NPs were observed to alleviate the negative impact of UVB radiation Si-NPs were observed to be more effective than Si in alleviating the UVB impact on plants	Tripathi et al. (2017)
Nano-Si; chemical; 14, 50, and 200	250, 1000 mg/L, seedlings, hydroponic	Nano-Si; chemical; 14, 50, and 200	*Arabidopsis thaliana*	Size-dependent uptake of silica by plants was observed A decrease in plant growth was observed with the size of 50 and 200 nm, which was attributed to a high negative zeta potential (and therefore a change in pH)	Slomberg and Schoenfisch (2012)
			Nontransgenic and Bt-transgenic cotton	A decrease in plant height and root and shoot biomass was observed in both transgenic and nontransgenic cotton NPs were transported from root to shoot via xylem Impacts on IAA concentration and CAT and SOD activity were observed under NP treatment	Le et al. (2014)

*TGA* thermogravimetric analysis, *ICP* inductively coupled plasma, *XRD* X-ray diffractometry, *FTIR* Fourier transform infrared spectroscopy

Silicon nanoparticles were observed to form a binary film at the epidermal cell wall after absorption, which may add structural color to plants (Strout et al. 2013). The impact was not limited to coloring; Si-NPs were also speculated to act as a strengthening material that may act as an agent to prevent fungal, bacterial, and nematodal infections and, thus, may increase disease resistance. The authors also concluded that a nano-silicon layer may reduce plant transpiration and, thus, make plants more resistant to drought, high temperature, and humidity.

In most studies, Si-NPs were observed to be beneficial to or ineffective for plants by either supporting plant growth or having no impact (Bao-shan et al. 2004; Suriyaprabha 2014; Siddiqui and Al-Whaibi 2014), except for a few reports where Si-NPs were observed to have a negative impact on plants (Slomberg and Schoenfisch 2012; Le et al. 2014). Slomberg and Schoenfisch (2012) reported that the toxic impact of Si-NPs was due to the change in pH in growth media that occurred due to the addition of Si-NPs. The author observed that, after excluding the change in pH, Si-NPs were ineffective for *Arabidopsis*. A study by Le et al. (2014) did not study zeta potential or pH maintenance, because the authors lacked information about the properties of Si-NPs, which raises questions about the phytotoxicity of Si-NPs.

## Use of Si-NPs in agriculture

The unique physiochemical properties of nanoscale silicon particles have useful applications in different sectors, including promising applications in the agricultural sector. The unique properties of Si-NPs allow them to cope with agricultural damage that may occur through climate change and/or abiotic stress (Tripathi et al. 2012). The application of Si-NPs in agriculture may also lead to global food security by helping in the development of improved varieties with high productivity (Parisi et al. 2015). Silicon nanoparticles are promising and have agricultural implications, and several new applications are being investigated for plants. In the agricultural sector, Si-NPs were observed to be applied as a weapon against heavy metal toxicity (Cui et al. 2017), UVB stress (Tripathi et al. 2017), salinity stress (Abdel-Haliem et al. 2017), dehydration (Jullok et al. 2016), etc. Moreover, additional novel applications of Si-NPs include their use as fertilizers, pesticides, and herbicides. Therefore, Si-NPs have the potential to improve crops for sustainable agriculture.

## Si-NPs as pesticides

In the present decade, nanotechnology has intervened in helping develop disease-free agricultural crops (Gogos et al. 2012). Under the large umbrella of nanotechnology, nanosilica, a unique type of nanomaterial, is used as a nanopesticide. A number of studies point towards the use of Si-NPs as nanopesticides (Ulrichs et al. 2005; Rouhani et al. 2012; El-Bendary and El-Helaly 2013; Magda and Hussein 2016; Ziaee and Ganji 2016). Si-NPs have been observed to be used in two ways: either Si-NPs were directly applied in the field and played the role of pesticides, killing insects and larvae, or mesoporous silica nanoparticles were used as nanocarriers that released commercial pesticides to enhance their efficiency. It was shown that SiO_2_ NPs had lethal properties for *Callosobruchus maculatus* (Rouhani et al. 2012); it was observed that the nanoparticles were more effective on adult insects than larvae, and it was speculated that the impact could be due to dehydrating properties of silica, which may result in impairment of the digestive tract or surface enlargement of the integument. The lethal impact of Si-NPs for pests can also be due to blockage of spiracles and tracheas or damage to the protective wax coating on the cuticle by sorption and abrasion. Some related and similar impacts of Si-NPs on different insects are listed in Table 2. A surface-charged modified hydrophobic nanosilica (∼ 3 to 5 nm) demonstrated the potential to eradicate an array of crop insects, pests, and animal ectoparasites of veterinary importance (Ulrichs et al. 2005). The working mechanism by which nanosilica controls pests was speculated to be breaking the protective lipid water barrier by physisorption of nanosilica, which resulted in the death of targeted organisms (Ulrichs et al. 2005; Rai and Ingle 2012). To improve the efficacy of pesticides, it is imperative to release the pesticide at the targeted site. However, for the controlled release of bioformulations, mesoporous silica nanoparticles are essential to deliver pesticides (Li et al. 2007; Chen et al. 2011). Several reports have shown that mesoporous nanosilica increased the durability of commercial pesticides and their efficiency (Table 2). The studies clearly indicated that the Si-NPs are effective pesticides and can be used either alone or to facilitate the delivery of the other commercial pesticides.

**Table 2:** The role of Si-NPs as pesticides

Composition; Size (nm)	Concentration; species effective against	Impact	References
SiO_2_; 20–60	1, 1.5, 2, and 2.5 g/kg; *Callosobruchus maculatus*	Kills insect and larvae in a dose-dependent manner Useful in protection of stored grain	Rouhani et al. (2012)
Si-NPs; (-)	200, 300, 400, and 500 ppm; *Spodoptera littoralis*	Kills larvae in a dose-dependent manner Increases plant longevity and number of leaves per plant after 15 days of application	El-Helaly et al. (2016)
SiO_2_; 12, 20–30	50, 100, 200, and 300 ppm; *Rhyzopertha dominica, Tribolium confusum*	Kills insects in a dose- and size-dependent manner The impact of NPs was observed to be more intensive on wheat grains than barley grains Useful in protection of stored grains	Ziaee and Ganji (2016)

Composition	Encapsulated pesticide	Benefits	References
Si-NPs; thickness ~ 15 nm, surface area ~ 588 m^2^ g^−1^, pore diameter 4–5 nm	Avermectin	Increased photostability of pesticide and sustained release.	Li et al. (2007)
Si-NPs; surface area 822 m^2^ g^−1^, pore diameter 2.4 nm	Pyoluteorin	Sustained release (85.13% within 28 days) Increased antifungal activity Pesticide degrades in alkaline conditions, but when encapsulated in Si-NPs, it is not degraded.	Chen et al. (2011)

## Si-NPs as a delivering agent for herbicides and fertilizers

Due to the unique physical and chemical properties of silicon nanoparticles, they can easily enter into plant cells and affect the plant growth and development by affecting their metabolism through diverse interactions, thereby triggering the potential to combat stress conditions. In the present scenario, where the focus is to increase crop productivity or to eradicate weeds, Si-NPs may act as an agent for target-specific delivery of herbicides and fertilizers (Wanyika et al. 2012). Silicon nanocarriers have been observed to carry herbicides (chloroacetanilide, anilide, and benzimidazole) embedded in a diatom fistule and deliver the herbicide to the field in its active form (Lodriche et al. 2012). In the case of fertilizer delivery, studies signified that the application of nano-silicon dioxide with organic fertilizer was used to improve plant productivity (Janmohammadi et al. 2016). Mesoporous silica nanoparticles (MSNs) with a specific pore size (2–10 nm) served as an efficient delivery vector for urea-, boron-, and nitrogenous-based fertilizers (Torney et al. 2007; Wanyika et al. 2012) (Table 3). Thus, Si-NPs have the potential to be used as a fertilizer alone for specific crops and can be used to deliver herbicides and fertilizers in plants.

**Table 3:** Si-NP as fertilizers

Composition	Herbicide/fertilizer	Benefits	References
SiO_2_ NP, Size 20–30 nm, 180–600 m^2^ g^−1^	Farmyard manure (FYM), and NPK fertilizers (applied with 20 mM SiO_2_ NP)	The fertilizers were observed to be significantly effective to improve growth traits in presence of NP	Janmohammadi et al. (2016)
Si-NP; surface area (~ 1000 m^2^ g^−1^); pore size (25 nm)	Urease (encapsulated)	Adsorption of urease increased Enhancement in stability of urease was observed after adsorption May act as useful model for nitrogen release in soil	Hossain et al. (2008)
Si-NP; surface area (1013 m^2^ g^−1^); pore size (2.5 nm)	Urease (encapsulated)	A burst release of entrapped urea was observed within 24 h; however, slow release was observed in subsequent period Release profile was slower in soil than in the water	Wanyika et al. (2012)

## Si-NPs in target-specific delivery of proteins, nucleotides, and chemicals in plants

The implementation of several nanoplatforms in various fields under in vitro conditions has spawned concerns in agri-nanotechnology. This technology embraces the promise of controlled and regulated release of agrochemicals and site-targeted delivery of various macromolecules, such as proteins, nucleotides, and chemicals, for improved plant resistance and nutrient efficiency, as well as increased crop yields (Nair et al. 2010). Nanoencapsulation has demonstrated the efficient and protected use of chemicals with less release to the environment and ensures eco-protection (Tsuji 2001; Boehm et al. 2003). The uptake, competence, and impact of various nanoparticles on plant growth, development, and biochemical process vary diversely among various plant species. Specifically, the use of MSNs in targeted delivery of various chemicals provides new insight into the safe use of this novel technology for improving crop variety and yield. Mesoporous silicon nanoparticles have chemically and thermally stable structures with large surface areas, tunable pore sizes, and several well-characterized surface properties, which makes them suitable for hosting guest molecules (Torney et al. 2007). In addition, the size-adjustable 3D open pore structure allows the regulation of adsorption rates to create effective delivery carriers (Pérez-De-Luque and Rubiales 2009). Surface-coated MSNs were useful for DNA and SiRNA delivery due to their binding affinity and high cellular uptake (Xia et al. 2009). Likewise, Torney et al. (2007) demonstrated the transportation of DNA and chemicals (with the gene and its chemical inducer) into isolated plant cells and intact leaves through MSNs. The direct delivery of a Cre recombinase protein through gold-plated MSNs was also successfully achieved by a biolistic method in maize (Martin-Ortigosa et al. 2014). Therefore, MSNPs have become established as transportation materials and have the potential to be used for the development of genetically modified crops.

## Si-NPs as a component of nanozeolite for the purpose of increasing water holding capacity

Soil is the most essential factor that regulates plant growth by controlling nutrient and water reserves (Ghaemi et al. 2014); therefore, improved soil quality is vital for increasing crop productivity (Lal 2015). Natural zeolites are an important alternative to overcome the effects of drought in arid regions (Ghanbari and Ariafar 2013). Considering the increasing interest in this area, various nanosized zeolites have been prepared and used to improve soil quality, as well as the impacts of chemical and organic fertilizers, for crop improvement (Najafi-Ghiri 2014). To combat negative hydric impacts in soil, nanozeolites act as a slow-release source for water and, therefore, increase the water holding capacity of the soil (Sekhon 2014). Mirzaei et al. (2015) reported the effect of the application of nanozeolite and zeolite on the water-stable mean weight diameter (MWDw), which is an index of aggregation, stability and strength as well as the aggregate size fraction of carbon. The aggregation process plays a substantial role in improving soil physical characteristics, such as water conduction, infiltration, and ventilation. These zeolites and nanozeolites facilitate water infiltration and retention in the soil due to their porous and capillary properties. Zeolites are known to act as natural wetting agents and work as water distributors throughout the soil, ultimately affecting water conduction in plants (Szerment et al. 2014; Ghazavi 2015). Thus, the observed results clearly showed the ability of Si-NPs to enhance the water holding capacity and, therefore, improve soil quality.

## Si-NPs as nanosensors

In recent decades, environmental sensing technologies based on metallic nanoparticles have gained enormous attention due to their extraordinary sensing resolution and sensitivity to analytes under optical detection. Developing a nanoparticle-based sensing device has been proposed due to the surface area of nanoparticles and the availability of surface anchored bioactive molecules for analyte detection (Hache et al. 1986). Although functionalized nanoparticles such as silver, gold, and Eu_2_O_3_ have been broadly engaged to increase sensory power due to their surface-enhanced plasmon resonance, with these nanoparticles, there is a risk of altering the surface plasmon resonance spectrum (Jin et al. 2001; Jean et al. 2010). Silica nanoparticles were successfully used as nanosensors for the detection of various metals in soil. The core–shell structure of silica nanoparticles provides immense advantages over the other nanoparticles in terms of sensor stability, accuracy, and sensitivity (Sun et al. 2016). When combined with the other nanoparticles, silicon has the potential to improve stability and sensitivity; by firmly attaching well-separated silver nanoparticles on the silica surface, optical stability and sensitivity in suspensions are improved, and the thin-film structural configuration acts as a colloidal stabilizer and a spacer for the spatial stabilization of silver nanoparticles (Jean et al. 2010). Si-NPs with silver nanospheres (SiO_2_@Ag) were successfully employed as an optical sensor for melamine detection. Similarly, Liu et al. (2014) prepared rhodamine B-doped silica nanoparticles coated with carbon dots and used them as nanosensors for the ratiometric fluorescence imaging of copper ions from tap water. 8-Aminoquinoline-functionalized silica nanoparticles have been shown to be used as fluorescent nanosensors for the analysis of divalent zinc ions in aqueous and yeast cell suspensions (Rastogi et al. 2011). Therefore, the discussed studies clearly indicate that Si-NPs alone or in combination with the other nanoparticles act as effective sensors with potential for agricultural use.

## Conclusion and future perspectives

Nanotechnology is a promising area of interdisciplinary research that opens avenues in several fields, such as medicine, pharmaceuticals, electronics, and agriculture. This article presents the potential of Si-NPs in agriculture and brings together the literature relevant to the use of nanoparticles as pesticides, fertilizers, herbicides, genetic and drug transfer agents, soil improving agents, and sensors for soil analysis. Studies show that Si-NPs have the potential to revolutionize the existing technology used in various sectors, such as agriculture and plant biotechnology. Silicon nanoparticle-mediated targeting of biomolecules would be useful for developing new cultivars that are resistant to various biotic and abiotic factors. These nanoparticles can provide green and eco-friendly alternatives to various chemical fertilizers without harming nature. Thus, Si-NPs may have concrete solutions to many agricultural problems regarding weeds, pathogenicity, drought, crop yield, and productivity.
